# Point of care obstetric ultrasound knowledge retention among mid-wives following a training program: a prospective cohort pilot study

**DOI:** 10.1186/s12884-023-05429-4

**Published:** 2023-02-09

**Authors:** Aloysius G. Mubuuke, Rita Nassanga

**Affiliations:** Radiology Department, School of Medicine, College of Health Sciences, Makerere University, Kampala, Uganda

**Keywords:** Point of Care Obstetric Ultrasound, Midwives, Knowledge retention

## Abstract

**Background:**

Obstetric ultrasound has become a routine part of antenatal care in many parts of the world including low income settings. However, there is a shortage of radiologists and sonographers to perform routine obstetric scans in many areas especially in the rural settings of low income countries, despite having equipment available to do this. As a result, Point of care ultrasound (POCUS) has been suggested to bridge this gap by training other health workers such as midwives to perform basic obstetric ultrasound as part of their clinical care.

**Methods:**

It was a prospective cohort pilot study in which trained midwives in point of care obstetric ultrasound were followed up at 6 months post training to assess their knowledge retention. Eleven trained midwives were purposively selected and followed up for knowledge retention. These were trained for 6 weeks and were given a knowledge assessment immediately after training, then given an assessment at 6 months following training. Data was analyzed using SPSS. Wilcoxon signed rank test was used to compare assessments and perceived knowledge as well as Spearman correlation to test the relationship between the number of scans performed and exam assessments, knowledge and exam assessments, and number of scans and knowledge.

**Results:**

There were eleven midwives, all female with an average age of 42.3 years. The mean exam score (out of 50) was 44.2 at the end of the training and 42.9 at 6-months follow up. The midwives demonstrated higher perceived knowledge at the end of the training when compared to the 6-months follow up. However, this perceived higher knowledge was not statistically significant when correlated with the exam scores either at the end of the training or at the follow up of 6 months.

**Conclusion:**

This pilot study has demonstrated that training midwives in point of care obstetric ultrasound can result into acceptable levels of knowledge retention that assist the midwives to apply this knowledge when making routine clinical decisions in relation to pregnant women.

## Introduction

Point of care ultrasound (POCUS) has become an integral part in the management of obstetric cases across the globe [1–3]. This has been due to the relatively low numbers of radiologists and sonographers that are formally trained to perform comprehensive obstetric ultrasound scans [1]. Therefore, there has been a need to transfer some of the basic obstetric ultrasound skills and knowledge to other health professionals directly responsible for looking after pregnant women such as midwives. POCUS refers to the practice of other health professionals who are not imaging experts using ultrasound to diagnose basic emergency cases without waiting for a qualified imaging professional [3]. POCUS in obstetric situations has become one of the most important application of role extension to have midwives acquire key knowledge and skills to perform emergency obstetric scans during their routine clinical duties. This improves and quickens the management of these mothers.

POCUS is a fast method used by health workers to assist them to make quick clinical decisions during routine clinical practice, and it has been implemented in many settings [1–5]. In many low income settings, there are usually few radiologists and sonographers especially in rural based health facilities to perform obstetric ultrasound scans. This leads to many pregnant women seeking these services to wait for long times or resort to seeking the service from private health facilities. In some situations, they are referred to large urban health care facilities for obstetric scanning. This, however, may delay management. It has thus been argued that training midwives in basic point of care obstetric ultrasound would bridge this shortage in the short term. Some basic obstetric ultrasound parameters that are assessed during antenatal care can thus be transferred to the attending midwives. Previous literature has documented the positive role of POCUS [6–8]. The aim is to transfer some of the key basic ultrasound skills to other health workers to accelerate patient management.

POCUS training for non-radiology health care providers has taken root in various settings [9, 10]. In Kenya, a POCUS training program was successfully introduced in 2013 to address the rural health work force shortage in performing ultrasound scans [6]. In this study, it was reported that it is useful to have focused training with well-defined outcomes to increase the proficiency of other health workers in using ultrasound during their routine clinical work. Despite the fact that Uganda has started advocating for such training programs to be introduced especially for rural-based midwives that routinely manage pregnant women, there is a scarcity of published literature from the context of low income settings reporting the knowledge retention levels of trainees when longitudinally followed up over a defined period of time.

Various health professionals have been trained in POCUS obstetric ultrasound including those from low income countries [3]. However, the extent of knowledge retention among midwives especially from low income settings where POCUS has taken root has not been widely determined. This is very crucial because knowledge and skills acquisition might be high initially, but may decay overtime which eventually affects the application of what has been learnt. This has been reported to occur in many educational interventions. In this study, our working hypothesis was that a dedicated 6-week training course in POCUS obstetric ultrasound among midwives who handle obstetric cases can allow the trained midwives to retain this knowledge and apply the knowledge 6 months later. This was a pilot study whose purpose was to assess retention of knowledge and its application after 6 months following POCUS training among midwives at a rural hospital in Uganda.

## Methods

### Design and participants

This was a prospective cohort study in which the midwives trained in POCUS obstetric ultrasound were followed 6 months post training to assess their knowledge retention as the outcome. Eleven midwives who consented, were selected to participate in this pilot study. Purposive sampling was used to select the midwives. The inclusion was that a participant had to be a midwife because these are the people who mostly handle the pregnant mothers at the hospital including deliveries before doctors intervene. In addition, they should not have been trained in POCUS before. Midwives with prior POCUS training were excluded from the study as their prior knowledge would confound the findings. Only those who consented to be part were included in the study. The midwives stay at the hospital premises and therefore would come into the training sessions. The Principal Nursing Officer assisted to inform the midwives and also obtaining them to be part of the study.

#### POCUS training

A POCUS curriculum was developed in obstetric ultrasound by a panel of experts who agreed on the final competencies. The experts included 4 radiologists and 3 sonographers. The key competencies that were targeted included: identification of a fetus, identification of number of fetuses, identifying a fetal heartbeat, demonstrating fetal lie and presentation, localizing the placenta, assessing maternal cervical length, assessing amniotic fluid adequacy, estimating fetal age using Mean Sac Diameter (MSD), Crown Rump Length (CRL) & Biparietal Diameter (BPD). The training, which was entirely physical attendance was for 6 weeks and had both didactic and practical sessions. There were 6 didactic classes, one class each week. Each lesson lasted one and a half hours. Each week also had 2 h of practical demonstrations on a pregnant woman. After the 6 weeks, the participants were then free to apply the knowledge gained in POCUS during their clinical situations.

#### Data collection

Participants completed a questionnaire which captured the number of scans they had performed, a pre- and post-self-rated knowledge tool on a 10-point scale. The questionnaire tool used was formulated by the research team and was first piloted with 2 midwives before administering it to the rest of the participants. This is because instruments in literature were not very specific to the Ugandan setting as POCUS outcomes differ for different contexts. The tool contained information on number of scans performed and self-rated knowledge on the targeted outcomes as described in the POCUS curriculum above. They also completed an exam that comprised of 50 multiple choice questions. Of the 50 questions, 36 were written and 14 were video based. After 6 months, the trainees completed a follow up questionnaire as the first as well as the exam that contained similar content areas as the first one. Their self-rated knowledge of obstetric ultrasound and exam scores were then compared between end of the training and then 6 months following the training. The exam completed was set by the ultrasound experts. The exam questions also were centered around the targeted POCUS outcomes explained above.

### Analysis

Data was analyzed using SPSS (SPSS 24; IBM, Armonk, NY, USA, 2016). Statistics were provided for the end of training data as well as following 6 months after the training. Since this was a pilot study with just small numbers of trainees, we used non-parametric tests of association. In order to compare total, written and video exam assessments, as well as the perceived knowledge between end of training and following 6 months, the Wilcoxon signed rank tests were used. We used Spearman correlations to test the relationship between the number of scans performed and exam assessments, knowledge and exam assessments, and number of scans and knowledge.

### Ethics

Ethical approval to conduct the study was granted by Makerere University School of Health Sciences Research Ethics Committee (REC No. 2019–080). Additional administrative clearance to conduct the study was also obtained from Kiwoko Hospital.

#### POCUS training outcomes

The training was for a period of 6 weeks. The first two weeks comprised of didactic lectures on the basic principles of ultrasound and the last four weeks focused on practical obstetric ultrasound scanning with the patients. The participants were trained by both radiologists and sonographers. The targeted outcomes included: identification of a live fetus, identification of number of fetuses, identifying a fetal heartbeat, demonstrating fetal lie and presentation, localizing the placenta, assessing maternal cervical length, assessing amniotic fluid adequacy, estimating fetal age using Mean Sac Diameter (MSD), Crown Rump Length (CRL) and Biparietal Diameter (BPD) and demonstrating professionalism and ethics during scanning.

## Results

The purpose of this study was to assess knowledge retention of midwives following POCUS training at Kiwoko hospital in Uganda. The entire POCUS training involved 25 nurses and midwives combined. However, for this pilot study of assessing knowledge retention at 6 months follow up, we purposively selected 11 midwives as they directly deal with pregnant women on daily basis. Therefore, the findings presented here relate to the 11 midwives that were followed up at 6 months post-training. However, to give an indication of Kiwoko Hospital parameters regarding staff numbers and obstetric volume, Table 1 summarizes this information. For this specific study, all the eleven midwives were female with an average age of 42.3 years.

**Table 1:** Kiwoko Hospital Characteristics

Item	Number
Mid-wives	48
Nurses	136
Medical Officers	15
Monthly Deliveries	300
Monthly obstetric emergencies	70

The mean exam score (out of 50) was 44.2 at the end of the training and 42.9 at 6-months follow up. The mean video exam scores (out of 14) for the two points in time were 12.4 and 11.6 respectively. The equivalent for the written exam scores (out of 36) were 32.1 and 31.6. The Wilcoxon signed rank test showed no statistical significance when the three comparisons were made (Video, *p* = 0.13; Written, *p* = 0.70; Total, *p* = 0.49).

At the end of the POCUS training, all the 11 midwives reported being competent with using obstetric ultrasound while at the end of 6 months, 9 out of 11 midwives reported that they were competent in using ultrasound. The midwives demonstrated higher perceived knowledge (out of 10) at the end of the training (7.9) when compared to the 6-months follow up (5.7), *p* = 0.002. However, this perceived higher knowledge was not statistically significant when correlated with the exam scores either at the end of the training (rho = 0.31, *p* = 0.33) or at the follow up of 6 months (rho = 0.16, *p* = 0.61).

On average, the number of obstetric scans completed during the training and following 6-months were 60 and 5 respectively. Table 2 shows the number of scans performed by each participant during the 6-week POCUS training while Table 3 summarizes the number of scans performed by each participant between the conclusion of the 6-weeks training and the endline period of 6 months. No correlation was noted between the number of scans performed during POCUS training and the post exam score (rho = 0.031, *p* = 0.91). However, a significant correlation was noted between the number of scans performed during the POCUS training and perceived knowledge gained at 6-months follow up (rho = 0.802, *p* = 0.004).

**Table 2:** Number of scans performed during 6-week POCUS training.

Participants	Number of scans performed
Participant 1	20
Participant 2	18
Participant 3	17
Participant 4	23
Participant 5	26
Participant 6	19
Participant 7	20
Participant 8	21
Participant 9	17
Participant 10	21
Participant 11	22

**Table 3:** Number of scans performed between conclusion of 6-week training and endline period of 6 months.

Participants	Number of scans performed
Participant 1	68
Participant 2	65
Participant 3	50
Participant 4	55
Participant 5	65
Participant 6	70
Participant 7	50
Participant 8	60
Participant 9	60
Participant 10	63
Participant 11	64

## Discussion

The purpose of this pilot study was to assess knowledge retention of midwives after going through POCUS training in obstetric ultrasound. The midwives do handle pregnant mothers on daily basis and we thus thought it was wise to follow these up to determine their application and retention of this ultrasound knowledge at 6 months. Previous studies have followed up POCUS trainees at 6 months to assess knowledge retention [2, 3]. The goal of POCUS is to transfer some of the basic ultrasound skills to non-imaging health professionals such that these skills assist them in making clinical decisions to manage their patients. This also addresses the critical shortage of imaging professionals in many settings especially low income settings [4]. In this study, the midwives demonstrated minimal decrease in knowledge even at 6 months follow up as can be evidenced in the exam scores. This finding is similar to what has been previously reported. For example, in one study following a 13-days ultrasound course in echocardiography and perioperative ultrasound, anaesthetic interns sustained similar exam scores at the end of the training and at 90 days later [11]. However, another study reported that the residents` ultrasound knowledge increased in 2.5 h following a training workshop in ultrasound, but this knowledge significantly decreased twelve months later [12].

Generally, when knowledge and skills are not frequently used, they tend to decay over time. In this study, the midwives` perception was that their ultrasound knowledge had decreased at 6 months post-training. However, this was not corroborated by the exam scores obtained between the two points in time. The likely explanation for this is multifactorial. First, is the idea of reinforcement and dispersed learning [13, 14]. This relates to the fact that perhaps the midwives incorporated the POCUS knowledge and skills gained into their routine clinical practice when managing obstetric cases at the hospital. They therefore had opportunities to constantly apply the knowledge and skills learnt hence leading to longer term retention. However, the other reason could be the proctored POCUS training that was used that led to long term retention. This kind of training has been reported in other ultrasound training initiatives [15]. In addition, the practical hands on training by the facilitators during the 6 weeks of training plus the learning materials left behind could have contributed to constant referral to these materials hence longer term knowledge retention.

Due to the shortage of radiologists and sonographers in many low income settings including Uganda where this study was conducted, there have been efforts to transfer some basic ultrasound skills to other health professionals especially in rural settings so that they are able to use ultrasound to inform some of their quick clinical decisions. Obstetric ultrasound has thus been identified as one area where POCUS involving midwives is crucial as part of antenatal care. Despite these efforts however, there has been no empirical evidence to show whether targeted POCUS actually works over a sustained period of time to achieve the intended objective. This pilot study has thus demonstrated that targeted POCUS training may yield the envisaged objective over time as the midwives followed up at 6 months demonstrated satisfactory retention of ultrasound knowledge.

In our cohort, those who completed more scans during the POCUS training had higher perceived knowledge at 6 months follow up. This means that frequent practice of ultrasound may result in attainment of clinical competency in scanning, self-efficacy and self-confidence, a key principle in learning [16]. This is accentuated by the fact that the trainees received adequate feedback from the trainers during the POCUS training, hence further improving their proficiency. The number of scans completed was not uniform across the midwives and there was no prescribed number of scans that each participant had to perform. Each participant performed the scans according to their need in their routine clinical work. In addition, the midwives had no prior training in POCUS, hence any knowledge retention noted in this study could readily be attributable to our training intervention. However, one cannot rule out the fact that since the midwives knew that they would be re-tested at 6 months, they perhaps put in more effort to master the knowledge and skills taught and this may not necessarily guarantee that this level of proficiency can be sustained even beyond the 6 months. The amount of training, duration and clinical experience during POCUS required to achieve adequate proficiency in ultrasound for non-imaging professionals is still a subject of debate [17, 18]. This coupled with a lack of proper standardized curriculum, regulation and supervision still pose a challenge around POCUS training. What is however clear is that adequacy of training should be tagged to competency-based assessment where clear learning outcomes are well defined in any POCUS curriculum [19]. This study has demonstrated that those trained can actually attain that level of knowledge retention to aid their clinical practice and we believe that findings from this study do contribute to the current debates surrounding POCUS training and knowledge retention, especially from the context of low income settings where POCUS has taken root, but with a dearth of published literature to provide evidence that it may work. Country-specific training in POCUS need to involve radiology professional bodies in setting up standards and supervision mechanisms for the trainees.

This pilot study did not set out to evaluate impact of POCUS training among midwives on health outcomes and indicators such as reduced referrals, reduced maternal mortality due to obstetric emergencies and others. This needs more time as the trainees continue to use POCUS to demonstrate real impact on obstetric health outcomes. As such, more studies following trainees over longer periods of time to demonstrate real impact on health indicators are recommended in future. In addition, from this study, there seems to have been optimal utilization of POCUS by the midwives as each of them at least performed more than 50 scans post training. This further indicates that POCUS can be utilized, but also points out that the need is great as many mothers do report to the facility for obstetric scans. This is also justified by the fact that the hospital receives a substantial number of obstetric cases as can be seen from Table 1. Although Kiwoko hospital where this study was conducted had fairly stable power supply, standby generators and fairly good numbers of staff, the demand for POCUS in many other low-resource settings can be affected by a number of factors such as power shortages and low numbers of health workers which should be put into consideration when designing POCUS programmes.

The small sample size used in this study is a limitation. Therefore, findings may not be generalizable. However, it should be noted that this was a pilot study which has thus yielded important evidence that can be used to scale up and design larger studies to inform the practice of POCUS training. In addition, having a control group would perhaps have increased the rigor of the study. Lastly, our 6 months follow up assessment did not involve real practical bedside ultrasound scanning exam to assess and compare self-reported efficacy and real technical ability. Due to technical reasons, we could not do a real bedside ultrasound practical exam. The focus of this study was mainly on ultrasound knowledge retention following POCUS training and not necessarily technical proficiency with the ultrasound machines at the bedside. However, previous literature has reported that exam scores could provide some level of prediction on how the trainee can perform in a practical exam. We thus do recommend further studies in many other settings that involve follow up of more trainees over a longer period of time, incorporate real practical bedside ultrasound scanning in the testing to compare self-reported efficacy and real technical ultrasound scanning ability and perhaps have designs that have a control group.

## Conclusion

This pilot study has demonstrated that intensive POCUS training in obstetric ultrasound for midwives involving both didactic and practical sessions can result into satisfactory retention of knowledge that could be applied in routine obstetric care by the midwives. More studies are needed to determine if this longer retention can result into real clinical proficiency and positive impact to the pregnant women.

## Data Availability

Data are available upon request to the corresponding author.
